# Supplementary material for: Anthocyanin Intake and Physical Activity: Associations with the Lipid Profile of a US Working Population
Source: Molecules. 2020 Sep 24;25(19):4398. doi: 10.3390/molecules25194398 (PMC7582364; doi:10.3390/molecules25194398)
Supplement: Supplementary file 1 [file molecules-25-04398-s001.zip › supplementary materials/Supplementary Table 1.pdf]

Table S1: Independent associations of anthocyanin intake (SD) and physical activity level with lipid profile measures (mg/dL).

	Anthocyanin intake (SD)		Physical activity level*	
**Lipid profile:**	**β (95%CI)**	**p-value**	**β (95%CI)**	**p-value**
Triglycerides				
Age, sex, and energy adjusted model (95% CI)	-9.13 (-17.76 to -0.50)	0.04	-8.14 (-12.48 to -3.79)	<0.001
Multivariable adjusted model 1 (95% CI) [a]	-7.69 (-16.16 to 0.79)	0.08	-5.75 (-10.23 to -1.28)	0.01
Multivariable adjusted model 2 (95% CI) [b]	-9.06 (-18.10 to -0.03)	0.05	-5.70 (-10.59 to -0.81)	0.02
Multivariable adjusted model 3 (95% CI)[c/d]	-7.73 (-16.83 to 1.37)	0.10	-5.54 (-10.40 to -0.67)	0.03
Total cholesterol				
Age, sex, and energy adjusted model (95% CI)	-2.19 (-6.80 to 2.42)	0.35	-2.69 (-5.03 to -0.34)	0.03
Multivariable adjusted model 1 (95% CI) [a]	-2.91 (-7.61 to 1.80)	0.23	-3.15 (-5.63 to -0.67)	0.01
Multivariable adjusted model 2 (95% CI) [b]	-2.16 (-7.13 to 2.80)	0.39	-2.06 (-4.75 to 0.62)	0.13
Multivariable adjusted model 3 (95% CI)[c/d]	-1.36 (-6.38 to 3.67)	0.60	-2.06 (-4.75 to 0.64)	0.13
HDL cholesterol				
Age, sex, and energy adjusted model (95% CI)	1.43 (0.09 to 2.76)	0.04	0.99 (0.31 to 1.67)	0.01
Multivariable adjusted model 1 (95% CI) [a]	1.23 (-0.05 to 2.52)	0.06	0.59 (-0.09 to 1.28)	0.09
Multivariable adjusted model 2 (95% CI) [b]	1.15 (-0.22 to 2.52)	0.10	0.59 (-0.15 to 1.34)	0.12
Multivariable adjusted model 3 (95% CI)[c/d]	1.23 (-0.16 to 2.62)	0.08	0.56 (-0.18 to 1.30)	0.14
LDL cholesterol				
Age, sex, and energy adjusted model (95% CI)	-1.75 (-5.83 to 2.33)	0.40	-2.34 (-4.42 to -0.27)	0.03
Multivariable adjusted model 1 (95% CI) [a]	-2.47 (-6.65 to 1.70)	0.24	-2.94 (-5.13 to -0.75)	0.01
Multivariable adjusted model 2 (95% CI) [b]	-1.56 (-5.97 to 2.85)	0.49	-1.99 (-4.38 to 0.39)	0.10
Multivariable adjusted model 3 (95% CI)[c/d]	-1.05 (-5.53 to 3.43)	0.65	-1.99 (-4.37 to 0.40)	0.10
LDL:HDL				
Age, sex, and energy adjusted model (95% CI)	-0.11 (-0.21 to -0.01)	0.04	-0.11 (-0.16 to -0.06)	<0.001
Multivariable adjusted model 1 (95% CI) [a]	-0.12 (-0.22 to -0.01)	0.03	-0.10 (-0.16 to -0.05)	<0.001
Multivariable adjusted model 2 (95% CI) [b]	-0.10 (-0.21 to 0.01)	0.07	-0.09 (-0.15 to -0.03)	0.004
Multivariable adjusted model 3 (95% CI)[b/c]	-0.09 (-0.20 to 0.02)	0.11	-0.09 (-0.14 to -0.03)	0.005
TG:HDL				
Age, sex, and energy adjusted model (95% CI)	-0.29 (-0.55 to -0.02)	0.04	-0.25 (-0.39 to -0.12)	<0.001
Multivariable adjusted model 1 (95% CI) [a]	-0.24 (-0.50 to 0.02)	0.08	-0.18 (-0.32 to -0.04)	0.01
Multivariable adjusted model 2 (95% CI) [b]	-0.29 (-0.57 to -0.01)	0.05	-0.19 (-0.34 to -0.04)	0.02
Multivariable adjusted model 3 (95% CI)[c/d]	-0.25 (-0.53 to 0.03)	0.08	-0.18 (-0.33 to -0.03)	0.02
Total cholesterol:HDL				
Age, sex, and energy adjusted model (95% CI)	-0.17 (-0.30 to -0.04)	0.01	-0.15 (-0.21 to -0.08)	<0.001
Multivariable adjusted model 1 (95% CI) [a]	-0.17 (-0.30 to -0.04)	0.01	-0.12 (-0.19 to -0.06)	<0.001
Multivariable adjusted model 2 (95% CI) [b]	-0.16 (-0.29 to -0.02)	0.02	-0.11 (-0.18 to -0.03)	0.004
Multivariable adjusted model 3 (95% CI)[c/d]	-0.14 (-0.27 to -0.01)	0.04	-0.10 (-0.18 to -0.03)	0.005

[*]Physical activity was assessed using a scale of 0-7 representing levels of physical activity ranging from none to running >10 miles/wk or spending >3 hrs/wk in comparable physical activity.

[a]Adjusted for age, sex, BMI, smoking status, education level, marital status, prevalent hypertension, dyslipidemia, and type 2 diabetes.

[b]Adjusted for age, sex, BMI, total energy intake, mMDS, smoking status, education level, marital status, multivitamin use, supplement use, sleep, prevalent hypertension, dyslipidemia, and type 2 diabetes.

[c]Adjusted for age, sex, BMI, total energy intake, mMDS, smoking status, education level, marital status, multivitamin use, supplement use, sleep, activity level, sitting, sedentary behavior, prevalent hypertension, dyslipidemia, and type 2 diabetes.

[d]Adjusted for age, sex, BMI, total energy intake, mMDS, smoking status, education level, marital status, multivitamin use, supplement use, sleep, anthocyanin intake, prevalent hypertension, dyslipidemia, and type 2 diabetes.

TG: triglycerides, HDL: high density lipoprotein cholesterol, LDL: low density lipoprotein cholesterol

Boldface indicates statistical significance (p<0.05)
