# Supplementary material for: Anthocyanin Intake and Physical Activity: Associations with the Lipid Profile of a US Working Population
Source: Molecules. 2020 Sep 24;25(19):4398. doi: 10.3390/molecules25194398 (PMC7582364; doi:10.3390/molecules25194398)
Supplement: Supplementary file 1 [file molecules-25-04398-s001.zip › supplementary materials/Supplementary Table 2.pdf]

Table S2. Self-Reported Physical Activity (SRPA) questionnaire.

Physical Activity in the Past Month. Below circle ONE of the values (0 to 7) which best represents your general ACTIVITY LEVEL for the PREVIOUS MONTH.

**0-1: I did not participate regularly in programmed recreation, sport, or heavy physical activity.**	
0 -	Avoid walking or exertion (as an example, always use elevator, drive whenever possible instead of walking, biking or rollerblading).
1 -	Walk for pleasure, routinely use stairs, occasionally exercise sufficiently to cause heavy breathing or perspiration.
**2-3: I participated regularly in recreation or work requiring modest physical activity, such as golf, horseback riding, calisthenics, gymnastics, table tennis, bowling, weightlifting, yard work.**	
2 -	10 to 60 minutes per week.
3 -	Over one hour per week.
**4-7: I participated regularly in heavy physical exercise such as running or jogging, swimming, cycling, rowing, skipping rope, running in place or engaging in vigorous aerobic activity type exercise such as tennis, basketball or handball.**	
4 -	Run less than 1 mile per week or spend less than 30 minutes per week in comparable physical activity.
5 -	Run 1 to 5 miles per week or spend 30 to 60 minutes per week in comparable physical activity.
6 -	Run 5 to 10 miles per week or spend 1 to 3 hours per week in comparable physical activity.
7 -	Run over 10 miles per week or spend over 3 hours per week in comparable physical activity.
