# Supplementary material for: Anthocyanin Intake and Physical Activity: Associations with the Lipid Profile of a US Working Population
Source: Molecules. 2020 Sep 24;25(19):4398. doi: 10.3390/molecules25194398 (PMC7582364; doi:10.3390/molecules25194398)
Supplement: Supplementary file 1 [file molecules-25-04398-s001.zip › supplementary materials/Supplementary Table 3.pdf]

Table S3. Description of the mMDS score developed using Feeding America's Bravest diet and lifestyle questionnaire.
The possible responses indicated in the top row are matched with their corresponding points in the row directly below.
Calculation of the modified Mediterranean diet score (mMDS):
mMDS1+(mMDS2+mMDS3)+mMDS4+mMDS5+mMDS6+mMDS9+mMDS12+mMDS15+mMDS16+(mMDS7+mMDS10+mMDS13)*(1-fh) +(mMDS8+mMDS11+mMDS14)*fh
[1] Weighted by the proportion of meals at home relative to the total number of meals per week (breakfast + lunch + dinner)
[2] Weighted by the proportion of meals at the firehouse (or on work time) relative to the total number of meals per week (breakfast + lunch + dinner)

**mMDS items**	**Components**	**Score range**	**Points for each answer**								
1. Fast-food or Take-out food	How many times per week do you eat the following?	0-4	never	≤ 1	2-3	3-4	5-6	every day	8-10	≥ 11	missing
			4 pts	3 pts	2 pts	2 pts	1 pts	1 pts	0 pts	0 pts	0 pts
2. Fruits	How many servings of each of the following do you consume per day?	0-4	0	≤ 1	2-3	3-4	5-6	≥ 7			missing
			0 pts	1 pts	2 pts	3 pts	4 pts	4 pts			0 pts
3. Vegetables (not including potatoes)	How many servings of each of the following do you consume per day?	0-4	0	≤ 1	2-3	3-4	5-6	≥ 7			missing
			0 pts	1 pts	2 pts	3 pts	4 pts	4 pts			0 pts
4. Legumes (e.g. beans, chickpeas, lentils)	How many servings of each of the following foods do you eat per week?	0-4	0	≤ 1	2-3	3-4	5-6	≥ 7			missing
			0 pts	0 pts	1 pts	2 pts	3 pts	4 pts			0 pts
5. Nuts (e.g. walnuts, almonds, hazelnuts, pistachio, peanuts)	How many servings of each of the following foods do you eat per week?	0-4	0	≤ 1	2-3	3-4	5-6	≥ 7			missing
			0 pts	0 pts	1 pts	2 pts	3 pts	4 pts			0 pts
6. Sweet Desserts (cake, cookies, pie, ice cream, etc.)	How many times per week do you eat the following?	0-4	never	≤ 1	2-3	3-4	5-6	≥ 7			missing
			4 pts	4 pts	3 pts	2 pts	1 pts	0 pts			0 pts
7. Primary cooking oil/fat use at home[1]	Which oil or fat do you use most often for cooking and serving food at home?	0-5	Butter	Lard or other animal fat	Margarine	Corn or vegetable oil	Benechol or Smart Balance	Olive oil	EVOO	other	missing
			0	0 pts	1 pts	2 pts	3 pts	4 pts	5 pts	0 pts	0 pts
8. Primary cooking oil/fat use at work[2]	Which oil or fat do you use most often for cooking and serving food at the firehouse?	0-5	Butter	Lard or other animal fat	Margarine	Corn or vegetable oil	Benechol or Smart Balance	Olive oil	EVOO	other	missing
			0 pts	0 pts	1 pts	2 pts	3 pts	4 pts	5 pts	0 pts	0 pts
9. Fried foods (French fries, fried chicken, chicken nuggets, etc.)	How many times per week do you eat the following?	0-4	never	≤ 1	2-3	3-4	5-6	every day	8-10	≥ 11	missing
			4 pts	3 pts	2 pts	1 pts	0 pts	0 pts	0 pts	0 pts	0

10. Breads/starches consumed at home[1]	Which bread or starch do you most frequently eat at home?	0-4	I do not eat bread or starch	White bread, filled pasta, white rice, or potatoes	Durum wheat bread or dry pasta	French bread or Italian bread or multigrain or other crusty bread	Whole wheat bread or brown rice or whole wheat pasta	missing								
			3 pts	0 pts	3 pts	2 pts	4 pts	0 pts								
11. Breads/starches consumed at work[2]	Which bread or starch do you most frequently eat at the firehouse?	0-4	I do not eat bread or starch	White bread, filled pasta, white rice, or potatoes	Durum wheat bread or dry pasta	French bread or Italian bread or multigrain or other crusty bread	Whole wheat bread or brown rice or whole wheat pasta	missing								
			3 pts	0 pts	3 pts	2 pts	4 pts	0 pts								
12. Baked, broiled, grilled, or blackened (NOT fried) ocean fish (salmon, tuna, cod, haddock, etc.)	How many times per week do you eat the following?	0-4	never	≤ 1	2-3	3-4	5-6	every day	8-10	≥ 11	missing					
			0 pts	1 pts	2 pts	3 pts	4 pts	4 pts	4 pts	4 pts	0 pts					
13. Non-alcoholic beverages at home[1]	Which of the following non-alcoholic beverages do you most frequently drink at home?	0-4	Cola/ soda	Diet cola/soda	Fruit drink or punch	Milk	Tea/ coffee	Juice	Water	Other	missing					
			0 pts	1 pts	1 pts	1 pts	2 pts	2 pts	4 pts	0 pts	0 pts					
14. Non-alcoholic beverages at work[2]	Which of the following non-alcoholic beverages do you most frequently drink at the firehouse?	0-4	Cola/ soda	Diet cola/soda	Fruit drink or punch	Milk	Tea/ coffee	Juice	Water	Other	missing					
			0 pts	1 pts	1 pts	1 pts	2 pts	2 pts	4 pts	0 pts	0 pts					
15. Quantity of alcoholic beverages	How many alcoholic beverages (beer, wine, hard liquor, etc.) do you drink over a typical week?	0-4	I do not drink	0	1-2	3-4	5-6	7-8	9-10	11-12	13-14	15-16	17-18	19-20	≥ 21	missing
			0 pts	0 pts	2 pts	2 pts	4 pts	4 pts	4 pts	4 pts	4 pts	4 pts	4 pts	4 pts	1 pts	0 pts
16. Wine consumption	When you drink alcoholic beverages, what type do you drink?	0-2	White wine	Red wine	Beer	Hard liquors	Don’t drink									
			2 pts	2 pts	0 pts	0 pts	0 pts									
