# Supplementary material for: Anthocyanin Intake and Physical Activity: Associations with the Lipid Profile of a US Working Population
Source: Molecules. 2020 Sep 24;25(19):4398. doi: 10.3390/molecules25194398 (PMC7582364; doi:10.3390/molecules25194398)
Supplement: Supplementary file 1 [file molecules-25-04398-s001.zip › supplementary materials/Supplementary table 4.pdf]

Table S4: Sensitivity analysis for anthocyanin intake (SD) and physical activity level on lipid profile measures (mg/dL).

Sensitivity Analysis		Anthocyanin intake (SD)		Physical activity level*	
**Exclusions**	**n**	**β (95%CI)**	**p-value**	**β (95%CI)**	**p-value**
Chronic diseases[†a]	199				
Triglycerides		-9.09 (-19.24 to 1.06)	0.08	-5.89 (-11.64 to -0.14)	0.05
Total cholesterol		-2.29 (-7.67 to 3.12)	0.41	-0.55 (-3.62 to 2.52)	0.72
HDL cholesterol		0.92 (-0.59 to 2.44)	0.23	0.85 (-0.01 to 1.70)	0.05
LDL cholesterol		-1.44 (-6.14 to 3.27)	0.55	-0.83 (-3.50 to 1.84)	0.54
LDL:HDL		-0.09 (-0.21 to 0.03)	0.16	-0.08 (-0.15 to -0.01)	0.03
TG:HDL		-0.27 (-0.58 to 0.04)	0.09	-0.21 (-0.39 to -0.04)	0.02
Total cholesterol:HDL		-0.14 (-0.29 to 0.01)	0.07	-0.10 (-0.18 to -0.01)	0.03
Women[a]	236				
Triglycerides		-9.32 (-18.81 to 0.16)	0.05	-5.69 (-10.76 to -0.63)	0.03
Total cholesterol		-2.10 (-7.25 to 3.04)	0.42	-1.97 (4.72 to 0.77)	0.16
HDL cholesterol		0.72 (-0.67 to 2.12)	0.31	0.64 (-0.11 to 1.38)	0.09
LDL cholesterol		-1.01 (-5.58 to 3.55)	0.66	-1.97 (-4.40 to 0.46)	0.11
LDL:HDL		-0.08 (-0.20 to 0.03)	0.16	-0.09 (-0.15 to -0.03)	0.004
TG:HDL		-0.29 (-0.58 to 0.01)	0.06	-0.19 (-0.35 to -0.03)	0.02
Total cholesterol:HDL		-0.14 (-0.28 to 0.00)	0.05	-0.11 (-0.18 to -0.03)	0.004
Supplement use (proteins, glutamine, amino acids, etc.)[a]	170				
Triglycerides		-8.72 (-19.39 to 1.96)	0.11	-4.79 (-10.38 to 0.80)	0.09
Total cholesterol		-4.22 (-10.53 to 2.08)	0.19	-3.56 (-6.84 to -0.29)	0.03
HDL cholesterol		0.38 (-1.36 to 2.11)	0.69	0.23 (-0.68 to 1.14)	0.61
LDL cholesterol		-2.77 (-8.44 to 2.91)	0.34	-2.83 (-5.78 to 0.12)	0.06
LDL:HDL		-0.09 (-0.22 to 0.05)	0.21	-0.07 (-0.14 to -0.00)	0.04
TG:HDL		-0.21 (-0.51 to 0.10)	0.18	-0.10 (-0.26 to 0.06)	0.21
Total cholesterol:HDL		-0.13 (-0.29 to 0.03)	0.12	-0.09 (-0.18 to -0.01)	0.03

[*]Physical activity was assessed using a scale of 0-7 representing levels of physical activity ranging from none to running >10 miles/wk or spending >3 hrs/wk in comparable physical activity.

[†]Chronic diseases was defined as reporting a previous diagnosis or current treatment for hypertension, dyslipidemia, or diabetes, respectively.

[a]Adjusted for age, sex, BMI, total energy intake, mMDS, smoking status, education level, marital status, multivitamin use, supplement use, sleep, anthocyanin intake, prevalent hypertension, dyslipidemia, and type 2 diabetes

TG: triglycerides, HDL: high density lipoprotein cholesterol, LDL: low density lipoprotein cholesterol

Boldface indicates statistical significance (p<0.05)
